# (*E*)-3-Nitro-*N*′-(3-nitro­benzyl­idene)­benzohydrazide

**DOI:** 10.1107/S1600536812005466

**Published:** 2012-02-17

**Authors:** Xiao-Yan Li

**Affiliations:** a Zibo Vocational Institute, Zibo 255314, People’s Republic of China

## Abstract

In the title compound, C_14_H_10_N_4_O_5_, the mol­ecule exists in a *trans* conformation with respect to the methyl­idene unit. The dihedral angle between the benzene rings is 9.8 (2)°. In the crystal, mol­ecules are linked through N—H⋯O hydrogen bonds to form chains along the *c* axis.

## Related literature

For the syntheses and crystal structures of hydrazone compounds, see: Hashemian *et al.* (2011 ▶); Lei (2011 ▶); Shalash *et al.* (2010 ▶). For the crystal structures of similar compounds reported recently by the author, see: Li (2011*a* ▶,*b* ▶).
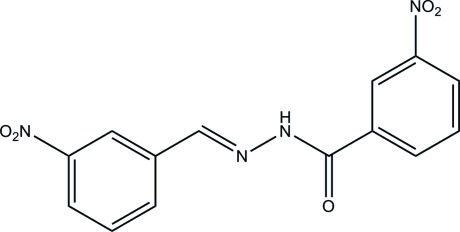


## Experimental

### 

#### Crystal data

C_14_H_10_N_4_O_5_
*M* *_r_* = 314.26
Monoclinic, 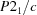

*a* = 11.990 (2) Å
*b* = 13.558 (3) Å
*c* = 8.5800 (17) Å
β = 96.752 (3)°
*V* = 1385.1 (5) Å^3^
*Z* = 4
Mo *K*α radiation
μ = 0.12 mm^−1^
*T* = 298 K
0.17 × 0.17 × 0.13 mm

#### Data collection

Bruker SMART CCD area-detector diffractometer
Absorption correction: multi-scan (*SADABS*; Sheldrick, 1996 ▶) *T*_min_ = 0.980, *T*_max_ = 0.985
9991 measured reflections
2549 independent reflections
1489 reflections with *I* > 2σ(*I*)
*R*_int_ = 0.094

#### Refinement

*R*[*F* ^2^ > 2σ(*F* ^2^)] = 0.078
*wR*(*F* ^2^) = 0.151
*S* = 1.03
2549 reflections
211 parameters
1 restraint
H atoms treated by a mixture of independent and constrained refinement
Δρ_max_ = 0.20 e Å^−3^
Δρ_min_ = −0.21 e Å^−3^

### 

Data collection: *SMART* (Bruker, 1998 ▶); cell refinement: *SAINT* (Bruker, 1998 ▶); data reduction: *SAINT*; program(s) used to solve structure: *SHELXS97* (Sheldrick, 2008 ▶); program(s) used to refine structure: *SHELXL97* (Sheldrick, 2008 ▶); molecular graphics: *SHELXTL* (Sheldrick, 2008 ▶); software used to prepare material for publication: *SHELXTL*.

## Supplementary Material

Crystal structure: contains datablock(s) global, I. DOI: 10.1107/S1600536812005466/rz2706sup1.cif

Structure factors: contains datablock(s) I. DOI: 10.1107/S1600536812005466/rz2706Isup2.hkl

Supplementary material file. DOI: 10.1107/S1600536812005466/rz2706Isup3.cml

Additional supplementary materials:  crystallographic information; 3D view; checkCIF report

## Figures and Tables

**Table 1:** Hydrogen-bond geometry (Å, °)

*D*—H⋯*A*	*D*—H	H⋯*A*	*D*⋯*A*	*D*—H⋯*A*
N3—H3⋯O3^i^	0.89 (1)	2.03 (2)	2.876 (4)	159 (4)

Symmetry code: (i) 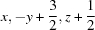.

**Fig. 1.**
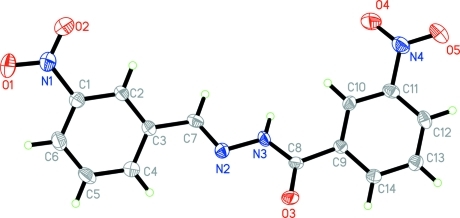
The molecular structure of the title compound, showing displacement ellipsoids drawn at the 30% probability level.

**Fig. 2.**
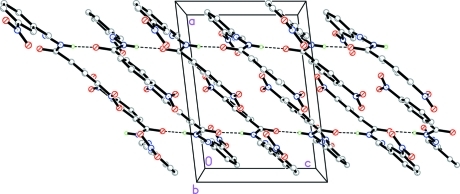
Molecular packing diagram of the title compound, viewed along the b axis. Hydrogen bonds are indicated by dashed lines. The C-bound H-atoms have been omitted for clarity.

## supplementary crystallographic information

### Comment

In recent years, hydrazone compounds have attracted much attention due to their syntheses and crystal structures (Hashemian *et al.*, 2011; Lei, 2011; Shalash *et al.*, 2010). As a continuation of our work on such compounds (Li, 2011*a*,*b*), the author reports herein on the crystal structure of the new title hydrazone compound.

The title compound (Fig. 1) exists in a *trans* configuration with respect to the methylidene unit. The dihedral angle between the C1–C6 and C9–C14 benzene rings of the molecule is 9.8 (2)°. The N1/O1/O2 and N4/O4/O5 nitro groups are tilted by 11.0 (2) and 15.5 (2)° with respect to the attached benzene rings. In the crystal, molecules are linked through N–H···O hydrogen bonds (Table 1) to form chains along the *c* axis (Fig. 2).

### Experimental

A mixture of 3-nitrobenzaldehyde (0.151 g, 1 mmol) and 3-nitrobenzohydrazide (0.181 g, 1 mmol) in 30 ml of ethanol containing few drops of acetic acid was refluxed for about 1 h. On cooling to room temperature, a solid precipitate was formed. The solid was filtered and then recrystallized from methanol. Yellow crystals, suitable for X-ray diffraction analysis, were obtained by slow evaporation of the solvent.

### Refinement

The amino H atom was located from a difference Fourier map and refined isotropically with the N—H distance restrained to 0.90 (1) Å. The remaining H-atoms were positioned geometrically and refined using a riding model, with C–H = 0.93 Å, and with *U*_iso_(H) set to 1.2*U*_eq_(C).

### Crystal data

**Table:**

C_14_H_10_N_4_O_5_	*F*(000) = 648
*M**_r_* = 314.26	*D*_x_ = 1.507 Mg m^−^^3^
Monoclinic, *P*2_1_/*c*	Mo *K*α radiation, λ = 0.71073 Å
Hall symbol: -P 2ybc	Cell parameters from 500 reflections
*a* = 11.990 (2) Å	θ = 2.7–24.5°
*b* = 13.558 (3) Å	µ = 0.12 mm^−^^1^
*c* = 8.5800 (17) Å	*T* = 298 K
β = 96.752 (3)°	Block, yellow
*V* = 1385.1 (5) Å^3^	0.17 × 0.17 × 0.13 mm
*Z* = 4	

### Data collection

**Table:**

Bruker SMART CCD area-detector diffractometer	2549 independent reflections
Radiation source: fine-focus sealed tube	1489 reflections with *I* > 2σ(*I*)
Graphite monochromator	*R*_int_ = 0.094
ω scans	θ_max_ = 25.5°, θ_min_ = 1.7°
Absorption correction: multi-scan (*SADABS*; Sheldrick, 1996)	*h* = −14→14
*T*_min_ = 0.980, *T*_max_ = 0.985	*k* = −16→16
9991 measured reflections	*l* = −10→10

### Refinement

**Table:**

Refinement on *F*^2^	Primary atom site location: structure-invariant direct methods
Least-squares matrix: full	Secondary atom site location: difference Fourier map
*R*[*F*^2^ > 2σ(*F*^2^)] = 0.078	Hydrogen site location: inferred from neighbouring sites
*wR*(*F*^2^) = 0.151	H atoms treated by a mixture of independent and constrained refinement
*S* = 1.03	*w* = 1/[σ^2^(*F*_o_^2^) + (0.0101*P*)^2^ + 0.8537*P*] where *P* = (*F*_o_^2^ + 2*F*_c_^2^)/3
2549 reflections	(Δ/σ)_max_ < 0.001
211 parameters	Δρ_max_ = 0.20 e Å^−^^3^
1 restraint	Δρ_min_ = −0.21 e Å^−^^3^

### Special details

**Table:**

Geometry. All e.s.d.'s (except the e.s.d. in the dihedral angle between two l.s. planes) are estimated using the full covariance matrix. The cell e.s.d.'s are taken into account individually in the estimation of e.s.d.'s in distances, angles and torsion angles; correlations between e.s.d.'s in cell parameters are only used when they are defined by crystal symmetry. An approximate (isotropic) treatment of cell e.s.d.'s is used for estimating e.s.d.'s involving l.s. planes.
Refinement. Refinement of *F*^2^ against ALL reflections. The weighted *R*-factor *wR* and goodness of fit *S* are based on *F*^2^, conventional *R*-factors *R* are based on *F*, with *F* set to zero for negative *F*^2^. The threshold expression of *F*^2^ > σ(*F*^2^) is used only for calculating *R*-factors(gt) *etc*. and is not relevant to the choice of reflections for refinement. *R*-factors based on *F*^2^ are statistically about twice as large as those based on *F*, and *R*- factors based on ALL data will be even larger.

### Fractional atomic coordinates and isotropic or equivalent isotropic displacement parameters (Å^2^)

**Table:**

	*x*	*y*	*z*	*U*_iso_*/*U*_eq_	
N1	0.8246 (4)	1.2678 (3)	0.8515 (5)	0.0664 (11)	
N2	0.8191 (3)	0.8283 (2)	0.9318 (3)	0.0395 (8)	
N3	0.7696 (3)	0.7597 (2)	1.0230 (3)	0.0406 (8)	
N4	0.4731 (3)	0.6122 (3)	1.3512 (4)	0.0570 (10)	
O1	0.8698 (3)	1.3372 (2)	0.7988 (5)	0.1044 (14)	
O2	0.7442 (4)	1.2775 (2)	0.9211 (6)	0.1224 (18)	
O3	0.7514 (2)	0.65035 (18)	0.8226 (3)	0.0499 (8)	
O4	0.4850 (3)	0.6965 (3)	1.3969 (4)	0.0972 (14)	
O5	0.4032 (3)	0.5562 (2)	1.3942 (4)	0.0755 (10)	
C1	0.8687 (3)	1.1684 (3)	0.8334 (5)	0.0452 (10)	
C2	0.8264 (3)	1.0930 (3)	0.9150 (4)	0.0410 (10)	
H2	0.7745	1.1060	0.9847	0.049*	
C3	0.8614 (3)	0.9976 (3)	0.8931 (4)	0.0350 (9)	
C4	0.9389 (3)	0.9806 (3)	0.7874 (4)	0.0455 (10)	
H4	0.9617	0.9164	0.7694	0.055*	
C5	0.9824 (3)	1.0578 (3)	0.7092 (5)	0.0522 (11)	
H5	1.0353	1.0454	0.6406	0.063*	
C6	0.9481 (3)	1.1527 (3)	0.7317 (5)	0.0526 (11)	
H6	0.9777	1.2051	0.6800	0.063*	
C7	0.8141 (3)	0.9163 (3)	0.9779 (4)	0.0407 (10)	
H7	0.7799	0.9301	1.0673	0.049*	
C8	0.7364 (3)	0.6733 (3)	0.9574 (4)	0.0342 (9)	
C9	0.6758 (3)	0.6059 (2)	1.0562 (4)	0.0304 (8)	
C10	0.6077 (3)	0.6410 (3)	1.1635 (4)	0.0356 (9)	
H10	0.6023	0.7083	1.1822	0.043*	
C11	0.5486 (3)	0.5746 (3)	1.2414 (4)	0.0399 (10)	
C12	0.5539 (3)	0.4742 (3)	1.2183 (4)	0.0425 (10)	
H12	0.5125	0.4309	1.2729	0.051*	
C13	0.6222 (3)	0.4398 (3)	1.1121 (4)	0.0407 (10)	
H13	0.6280	0.3723	1.0951	0.049*	
C14	0.6824 (3)	0.5053 (3)	1.0302 (4)	0.0359 (9)	
H14	0.7274	0.4817	0.9574	0.043*	
H3	0.759 (3)	0.772 (3)	1.1226 (19)	0.080*	

### Atomic displacement parameters (Å^2^)

**Table:**

	*U*^11^	*U*^22^	*U*^33^	*U*^12^	*U*^13^	*U*^23^
N1	0.085 (3)	0.043 (2)	0.076 (3)	−0.010 (2)	0.030 (2)	−0.001 (2)
N2	0.047 (2)	0.0364 (19)	0.0361 (18)	−0.0021 (16)	0.0085 (16)	0.0011 (15)
N3	0.054 (2)	0.0386 (19)	0.0323 (18)	−0.0114 (16)	0.0186 (17)	0.0019 (16)
N4	0.058 (2)	0.067 (3)	0.050 (2)	−0.010 (2)	0.022 (2)	−0.008 (2)
O1	0.134 (3)	0.042 (2)	0.150 (4)	−0.017 (2)	0.069 (3)	0.013 (2)
O2	0.149 (4)	0.048 (2)	0.192 (5)	0.016 (2)	0.114 (4)	0.008 (2)
O3	0.079 (2)	0.0418 (16)	0.0322 (15)	−0.0013 (14)	0.0225 (14)	−0.0069 (12)
O4	0.117 (3)	0.080 (3)	0.109 (3)	−0.023 (2)	0.073 (2)	−0.043 (2)
O5	0.067 (2)	0.084 (2)	0.083 (2)	−0.0112 (19)	0.0412 (19)	0.0048 (19)
C1	0.049 (3)	0.039 (2)	0.049 (3)	−0.005 (2)	0.012 (2)	−0.005 (2)
C2	0.041 (2)	0.041 (2)	0.044 (2)	−0.0053 (19)	0.0150 (19)	−0.0011 (19)
C3	0.032 (2)	0.040 (2)	0.032 (2)	−0.0038 (18)	0.0041 (18)	0.0009 (17)
C4	0.047 (3)	0.045 (3)	0.046 (2)	0.001 (2)	0.010 (2)	−0.002 (2)
C5	0.044 (3)	0.065 (3)	0.051 (3)	−0.008 (2)	0.021 (2)	−0.002 (2)
C6	0.058 (3)	0.053 (3)	0.049 (3)	−0.012 (2)	0.015 (2)	0.006 (2)
C7	0.042 (2)	0.050 (3)	0.032 (2)	0.005 (2)	0.0124 (18)	−0.0008 (19)
C8	0.038 (2)	0.034 (2)	0.032 (2)	0.0040 (18)	0.0119 (18)	−0.0011 (18)
C9	0.031 (2)	0.035 (2)	0.0251 (19)	−0.0016 (17)	0.0025 (16)	−0.0007 (16)
C10	0.040 (2)	0.032 (2)	0.035 (2)	−0.0057 (17)	0.0045 (18)	−0.0054 (17)
C11	0.037 (2)	0.054 (3)	0.029 (2)	−0.001 (2)	0.0058 (18)	−0.0064 (19)
C12	0.043 (3)	0.050 (3)	0.035 (2)	−0.009 (2)	0.0052 (19)	0.0112 (19)
C13	0.046 (2)	0.036 (2)	0.040 (2)	−0.0009 (19)	0.0027 (19)	0.0016 (18)
C14	0.037 (2)	0.040 (2)	0.030 (2)	0.0010 (18)	0.0031 (18)	−0.0014 (17)

### Geometric parameters (Å, º)

**Table:**

N1—O2	1.199 (4)	C4—C5	1.378 (5)
N1—O1	1.201 (4)	C4—H4	0.9300
N1—C1	1.463 (5)	C5—C6	1.372 (5)
N2—C7	1.261 (4)	C5—H5	0.9300
N2—N3	1.393 (4)	C6—H6	0.9300
N3—C8	1.340 (4)	C7—H7	0.9300
N3—H3	0.893 (10)	C8—C9	1.493 (5)
N4—O4	1.212 (4)	C9—C14	1.385 (5)
N4—O5	1.219 (4)	C9—C10	1.385 (4)
N4—C11	1.472 (5)	C10—C11	1.369 (5)
O3—C8	1.231 (4)	C10—H10	0.9300
C1—C2	1.370 (5)	C11—C12	1.378 (5)
C1—C6	1.382 (5)	C12—C13	1.376 (5)
C2—C3	1.379 (5)	C12—H12	0.9300
C2—H2	0.9300	C13—C14	1.387 (5)
C3—C4	1.391 (5)	C13—H13	0.9300
C3—C7	1.471 (5)	C14—H14	0.9300
			
O2—N1—O1	121.8 (4)	C5—C6—H6	120.9
O2—N1—C1	118.4 (4)	C1—C6—H6	120.9
O1—N1—C1	119.8 (4)	N2—C7—C3	121.2 (3)
C7—N2—N3	114.6 (3)	N2—C7—H7	119.4
C8—N3—N2	118.2 (3)	C3—C7—H7	119.4
C8—N3—H3	120 (3)	O3—C8—N3	123.1 (3)
N2—N3—H3	122 (3)	O3—C8—C9	120.9 (3)
O4—N4—O5	123.3 (4)	N3—C8—C9	115.9 (3)
O4—N4—C11	118.4 (4)	C14—C9—C10	119.7 (3)
O5—N4—C11	118.3 (4)	C14—C9—C8	118.0 (3)
C2—C1—C6	122.1 (4)	C10—C9—C8	122.1 (3)
C2—C1—N1	118.2 (4)	C11—C10—C9	118.5 (3)
C6—C1—N1	119.6 (4)	C11—C10—H10	120.7
C1—C2—C3	119.6 (3)	C9—C10—H10	120.7
C1—C2—H2	120.2	C10—C11—C12	123.0 (3)
C3—C2—H2	120.2	C10—C11—N4	118.5 (4)
C2—C3—C4	118.8 (3)	C12—C11—N4	118.5 (3)
C2—C3—C7	119.6 (3)	C13—C12—C11	118.1 (3)
C4—C3—C7	121.6 (3)	C13—C12—H12	120.9
C5—C4—C3	120.7 (4)	C11—C12—H12	120.9
C5—C4—H4	119.6	C12—C13—C14	120.2 (4)
C3—C4—H4	119.6	C12—C13—H13	119.9
C6—C5—C4	120.5 (4)	C14—C13—H13	119.9
C6—C5—H5	119.8	C9—C14—C13	120.4 (3)
C4—C5—H5	119.8	C9—C14—H14	119.8
C5—C6—C1	118.3 (4)	C13—C14—H14	119.8

### Hydrogen-bond geometry (Å, º)

**Table:**

*D*—H···*A*	*D*—H	H···*A*	*D*···*A*	*D*—H···*A*
N3—H3···O3^i^	0.89 (1)	2.03 (2)	2.876 (4)	159 (4)

Symmetry code: (i) *x*, −*y*+3/2, *z*+1/2.
